# Idiopathic granulomatous mastitis: Bridging multidisciplinary care through dermatologic expertise and treatment strategies

**DOI:** 10.1016/j.jdcr.2025.09.017

**Published:** 2025-09-26

**Authors:** Emily R. Nadelmann, Simran Randev, Christina M. Coyle, Caroline Halverstam

**Affiliations:** a Division of Dermatology, Montefiore Medical Center/Albert Einstein College of Medicine, Bronx, New York; b Department of Infectious Diseases, Albert Einstein College of Medicine, Bronx, New York

**Keywords:** breast, *Corynebacterium*, erythema nodosum, idiopathic granulomatous mastitis

## Case description

**Fig 1:**
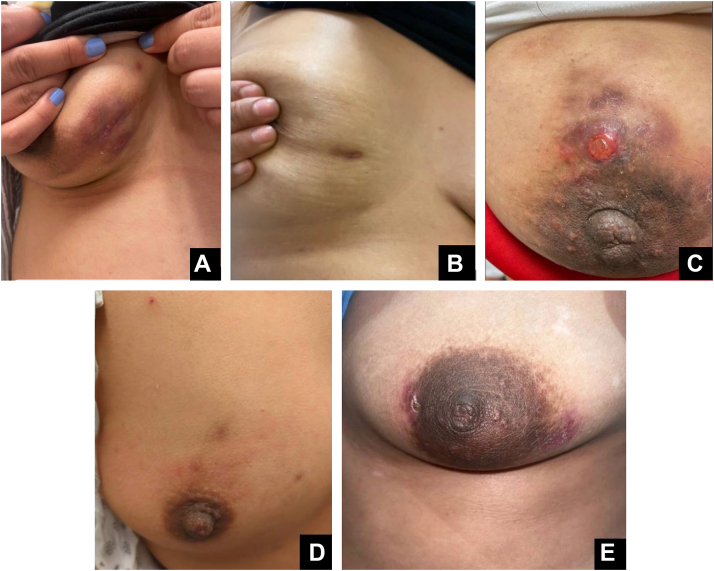
Clinical presentations in five women aged 25-40 years presenting to dermatology with breast involvement. **A,** Erythematous papules and nodules on the right inferior breast. **B,** Indurated nodulo-plaques with associated nipple retraction of the right breast. **C,** Erythematous, violaceous, and ulcerated dermal plaques with scale and crust around the left areola. **D,** Firm, indurated plaques involving the superior right breast. **E,** Crusted, indurated plaques with serous discharge along the right areola.

Five Hispanic women aged 24 to 40 presented to dermatology with idiopathic granulomatous mastitis (IGM), confirmed by ultrasound-guided core needle biopsy. Presentations included erythematous papules, indurated nodulo-plaques, ulcerated nodules with drainage, and nipple retraction. (Fig 1, *A-E*) One patient also presented with erythema nodosum. Cultures grew *Corynebacterium* species in 2 cases and *Staphylococcus epidermidis* in 1. Past medical histories included current pregnancy; prior latent tuberculosis treated with rifampin, isoniazid, pyrazinamide, ethambutol therapy; elevated prolactin; breast trauma; and vitiligo. All patients were co-managed by dermatology and infectious disease. Treatment was multimodal and tailored, involving intralesional corticosteroids, systemic corticosteroids, antibiotics such as doxycycline and trimethoprim-sulfamethoxazole, and immunomodulatory agents including methotrexate and adalimumab. Follow-up ranged from several months to 4 years, allowing monitoring of disease progression and treatment response (Supplementary Table I, available via Mendeley at https://data.mendeley.com/datasets/9d5bx9d5c9/1).

**Question: Which of the following treatments has not been shown to be helpful for IGM?**

A. Oral corticosteroids
B. Methotrexate
C. Doxycycline
D. Rituximab
E. Intralesional steroid injection

**Answer: D.** Rituximab.

## Discussion

IGM is a complex inflammatory breast disease with unclear pathogenesis. Proposed causes include autoimmune responses triggered by epithelial damage, inflammatory reactions to lobular secretions during pregnancy and breastfeeding, and trauma-induced immune cell migration.^1^ Our cases also included histories of pregnancy, elevated prolactin levels, treated latent tuberculosis, breast trauma, and autoimmune disorders, highlighting potential autoimmune, infectious, hormonal, and mechanical associations. Infectious agents, especially *Corynebacterium* species seen in 2 of our patients, may also contribute.^2^

Erythema nodosum, an inflammatory panniculitis with tender erythematous nodules on the shins, has been reported in association with IGM and was observed in 1 of our patients, reflecting possible shared autoimmune mechanisms.^3^

Clinically, patients present with painful, unilateral breast masses, including papules, nodules, ulcerated plaques, and nipple retraction.^1^ Most affected women are of childbearing age, often within 5 years postpartum, suggesting hormonal influences.^1^ Differential diagnosis requires exclusion of breast cancer, tuberculosis, sarcoidosis, and autoimmune diseases using purified protein derivative, chest imaging, prolactin testing, and ultrasound-guided core needle biopsy, which is the diagnostic gold standard.

Treatment strategies remain informed by small case series and clinical experience. In our cohort, intralesional corticosteroid was the most consistently effective intervention, often achieving resolution of localized inflammation. Systemic corticosteroids provided acute symptom control but were frequently followed by recurrence. Antibiotics such as doxycycline and trimethoprim sulfamethoxazole were variably effective and generally served as adjuncts. Immunomodulatory agents such as methotrexate and adalimumab were used in refractory or relapsing cases, where they contribute to clinical improvement. All of the above treatments have been reported in the literature as potential therapies for IGM; however, there are no reports of rituximab being used for IGM.^4^^,^^5^

Follow-up ranged from several months up to 4 years, highlighting the need for long-term monitoring and individualized care strategies. These observations are limited by the small sample size and underscore the need for larger, controlled studies to inform evidence-based treatment guidelines.

Dermatologists play a unique and essential role in managing granulomatous conditions like IGM given their expertise in inflammatory and granulomatous skin disorders. They are well-positioned to help lead the development of standardized diagnostic and treatment protocols, fostering multidisciplinary collaboration to optimize patient outcomes.

## Conflicts of interest

None disclosed.
